# Hiding in Plain Sight: an Approach to Treating Patients with Severe COVID-19 Infection

**DOI:** 10.1128/mBio.00398-20

**Published:** 2020-03-20

**Authors:** David S. Fedson, Steven M. Opal, Ole Martin Rordam

**Affiliations:** a 57, chemin du Lavoir, Sergy Haut, France; b Department of Medicine, Warren Alpert Medical School of Brown University, Providence, Rhode Island, USA; c Fyordgata 59, Trondheim, Norway

**Keywords:** COVID-19, endothelial dysfunction, generic drugs, host response treatment

## Abstract

Patients with COVID-19 infection are at risk of acute respiratory disease syndrome (ARDS) and death. The tissue receptor for COVID-19 is ACE2, and higher levels of ACE2 can protect against ARDS. Angiotensin receptor blockers and statins upregulate ACE2. Clinical trials are needed to determine whether this drug combination might be used to treat patients with severe COVID-19 infection.

## COMMENTARY

The severe respiratory disease that has recently emerged in China is caused by a novel coronavirus (COVID-19) (1). The virus is similar to the SARS coronavirus that spread internationally in 2003, infected more than 8,000 people, and killed almost 800. Infection with COVID-19 has now spread throughout the world, causing widespread social and economic disruption. To control its spread, Chinese officials have imposed extensive travel bans and quarantined large areas. Accelerated development of new vaccines and treatments is already under way. It is too early to know whether any of these efforts will contain the outbreak.

Thus far, patients hospitalized with severe COVID-19 infection have had pneumonia (2). Of 44,672 laboratory-confirmed patients, almost 5% have had critical illness and almost 50% of critically ill patients have died (3). The overall case fatality rate (2.3%) has been higher than that seen with seasonal influenza. Most deaths have involved older adults, many of whom have had underlying chronic illnesses. Although there is no known treatment for any coronavirus infection, investigators in China have undertaken several clinical trials. Except for corticosteroids, all of the drugs being tested target coronavirus replication. Unfortunately, very few of these antiviral drugs will be available to people who have been (or will be) infected with COVID-19. Yet, for those who develop severe disease, only one question matters: “will I live or die?” This is the question that clinical investigators should address. Could they discover a treatment that might reduce the severity of COVID-19 infection and improve patient survival?

In 2014, one of us suggested that statins might be used to treat patients with Ebola virus disease (4). A supply of a generic statin and a generic angiotensin receptor blocker (ARB) was sent to Sierra Leone. Experimental studies had shown that both drugs improved outcomes in experimental acute lung injury/acute respiratory disease syndrome (ARDS) (5–9). In Sierra Leone, local physicians treated approximately 100 Ebola patients with a combination of the two drugs. They noted “remarkable improvement” in survival. Although there was no support for a proper clinical trial, the findings from this unconventional and poorly documented treatment experience were published (10, 11). During the current Ebola outbreak in the Democratic Republic of the Congo (DRC), expensive vaccines are being used. Investigational monoclonal antibody preparations (12), but not inexpensive generic drug treatments (13), have been tested. There are preliminary signs that the DRC outbreak is coming under control, although the case fatality rate is still 66%.

An approach to treating patients with severe COVID-19 infection might be hiding in plain sight. The tissue receptor for the virus is ACE2, which is also the receptor for the SARS coronavirus (1). Several years ago, investigators in the Netherlands and elsewhere showed that ARBs and statins upregulate the activity of ACE2 (14, 15), and higher levels of ACE2 are associated with a reduced severity of ARDS (16). Both statins and ARBs target the host response to infection, not the virus (9). They act largely (although not exclusively) on endothelial dysfunction, which is a common feature of many virus infections (17). Both drugs counter endothelial dysfunction by affecting the ACE2/angiotensin-(1–7)/Mas and angiopoietin/Tie-2 signaling axes (9). Combination treatment with these two drugs appears to accelerate a return to homeostasis, allowing patients to recover on their own.

The host response is a major determinant of the pathogenesis of infectious diseases (18). We believe that investigators in China and elsewhere should undertake studies of patients with severe COVID-19 infection to determine whether targeting the host response with widely available and inexpensive generic drugs, like ARBs and statins, will improve their chances of survival. The studies would not have to be large; a successful clinical trial might require only 100 patients (9). Convincing evidence of the effectiveness of this treatment would suggest a syndromic approach to treating patients with other emerging infectious diseases, like Ebola and pandemic influenza, as well as everyday illnesses, like sepsis and pneumonia (19). The long-term benefits of these findings for global public health could be immense.
